# Common Pharmacophore of Structurally Distinct Small-Molecule Inhibitors of Intracellular Retrograde Trafficking of Ribosome Inactivating Proteins

**DOI:** 10.1038/srep03397

**Published:** 2013-12-02

**Authors:** Shichao Yu, Jewn Giew Park, Jennifer Nielsen Kahn, Nilgun E. Tumer, Yuan-Ping Pang

**Affiliations:** 1 Computer-Aided Molecular Design Laboratory, Mayo Clinic, Rochester, MN 55905, USA; 2 Department of Plant Biology and Pathology, School of Environmental and Biological Sciences, Rutgers University, New Brunswick, NJ 08901, USA

## Abstract

We reported previously (±)-2-(5-methylthiophen-2-yl)-3-phenyl-2,3-dihydroquinazolin-4(1*H*)-one [(±)-Retro-2^cycl^] as the chemical structure of Retro-2 that showed mouse protection against ricin, a notorious ribosome inactivating protein (RIP). Herein we report our chemical resolution of (±)-Retro-2^cycl^, analog synthesis, and cell-based evaluation showing that the two optically pure enantiomers and their achiral analog have nearly the same degree of cell protection against ricin as (±)-Retro-2^cycl^. We also report our computational studies explaining the lack of stereo preference and revealing a common pharmacophore of structurally distinct inhibitors of intracellular retrograde trafficking of RIPs. This pharmacophore comprises a central aromatic ring *o*-substituted by an aromatic ring and a moiety bearing an O or S atom attached to sp^2^ C atom(s). These results offer new insights into lead identification and optimization for RIP antidote development to minimize the global health threat caused by ribosome-inactivating proteins.

Ricin produced by the castor bean plant enzymatically depurinates the 28S rRNA and thereby functions as a ribosome-inactivating protein (RIP). This notorious RIP has been used repeatedly for bioterrorism^1^ as in letters sent to U.S. government officials in 2013. *Escherichia coli* produces Shiga-like toxins (Stx1 and Stx2; the latter being the most toxic^2^). Stx1 and Stx2 also function as RIPs and are responsible for food-borne outbreaks, including the deadliest outbreak in 2011^3^, with significant morbidity and mortality^4^. Presently there are no U.S. Food and Drug Administration approved vaccines or therapeutics to protect against ricin or other RIPs.

In an effort to minimize the global health threat caused by RIPs, we have been working on developing small-molecule RIP inhibitors that target the RIP catalytic domain using our doorstop approach^5^. In this endeavor we attempted to synthesize **Retro-2** (Figure 1) as a benchmark for our in vivo studies, because it was reported that 200 mg/kg **Retro-2** protected all inhibitor-treated mice against a dose of ricin that killed 90% of an unprotected control mouse population^6^. Serendipitously we discovered that the chemical structure of **Retro-2** is (±)-2-(5-methylthiophen-2-yl)-3-phenyl-2,3-dihydroquinazolin-4(1*H*)-one rather than (*E*)-2-(((5-methylthiophen-2-yl)methylene)amino)-*N*-phenylbenzamide^7^. The former is a racemic, cyclic compound abbreviated as **(±)-Retro-2^cycl^** (Figure 1), and the latter is an achiral, acyclic chemical (Figure 1). Because **(±)-Retro-2^cycl^** is a mixture of two stereoisomers, we set out to separate the mixture, determine the cell protection activities of the two enantiomers, and develop analogs of the active enantiomer(s) as a better benchmark for our in vivo studies.

Herein we report our chemical resolution of **(±)-Retro-2^cycl^**, synthesis of an achiral analog of **(±)-Retro-2^cycl^**, computational studies of **Retro-2^cycl^** isomers and other known inhibitors of intracellular transport of RIPs, and cell-based studies of **Retro-2^cycl^** isomers and their achiral analog. The results led to the development of a common pharmacophore of structurally distinct RIP transport inhibitors and offer important insight into RIP antidote development.

## Results

### Chemical resolution of (±)-Retro-2^cycl^

Although asymmetric syntheses^8^,^9^,^10^,^11^,^12^ have been reported for substituted 2,3-dihydroquinazolin-4(1*H*)-ones that are the scaffold of **Retro-2^cycl^**, we found that enantioselective reaction of 5-methylthiophene-2-carboxaldehyde with 2-aminobenzanilide using the readily available Sc(III)-inda-pybox as a catalyst^11^ gave the desired product with <10% yield and ~20% enantiomeric excess. Recognizing the presence of the aniline moiety in **Retro-2^cycl^**, we wanted to perform fractional crystallization of **(±)-Retro-2^cycl^** salts to be prepared from chiral acids. Unexpectedly, mixing **(±)-Retro-2^cycl^** with acids such as (1s)-(+)-10-camphorsulfonic acid caused decomposition of **(±)-Retro-2^cycl^** to 5-methylthiophene-2-carboxaldehyde and 2-aminobenzanilide, while weak acids such as (*R*)-(−)-mandelic acid, (−)-di-p-toluoyl-L-tartaric acid, (−)-dibenzoyl-L-tartaric acid, and (*D*)-(−)-tartaric acid did not form salts with **(±)-Retro-2^cycl^**. We eventually resorted to high-performance liquid chromatography (HPLC) separation using a chiral column. Although diastereomeric chiral stationary phases^13^ was reported for separation of thiazides that possess an aminal stereo center analogous to the one in **(±)-Retro-2^cycl^**, we used the Lux™ Amylose-2 chiral column from Phenomenex (Torrance, California) that has the 5-chloro-2-methylphenylcarbamate derivative of amylose as a chiral stationary phase^14^. Our rationale for using this type of stationary was that the phenyl rings of the column could form pi-pi interactions with the three aromatic rings of **(±)-Retro-2^cycl^** and that the carbamate NH group of the column would permit hydrogen bonds with the amide oxygen atom of **(±)-Retro-2^cycl^**. Indeed, using the Lux™ Amylose-2 chiral column, we readily separated **(±)-Retro-2^cycl^** as evidenced by the HPLC chromatogram showing two well-separated peaks with retention times of 9.350 and 17.517 minutes and areas under the peaks of 49.769% and 50.231%, respectively, under conditions described in Method (Figure 2). The 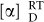 values of the two enantiomers are +118.7° and −126.5°, respectively. As described below, the cell protection studies showed that the two optically pure isomers demonstrated nearly the same degree of cell protection against ricin as the racemate. Therefore, we did not carry out the X-ray crystallography study to determine whether **(+)-Retro-2^cycl^** corresponds to **(*R*)-Retro-2^cycl^** or **(*S*)-Retro-2^cycl^**, but rather synthesized and tested an achiral analog of **(±)-Retro-2^cycl^**.

### Design and synthesis of an achiral analog of Retro-2^cycl^

**Figure 1:**
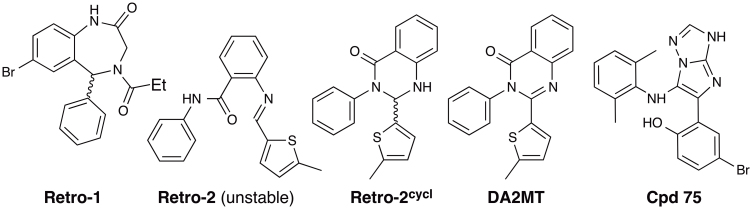
Chemical structures of Retro-2^cycl^, DA2MT, and other RIP transport inhibitors.

Energy minimizations at the HF/6-31G(d) level indicate that both enantiomers can adopt conformations at local minima of their potential energy surfaces (local minimum conformations). In those conformations the thiophene group is at the equatorial position of the heterocyclic ring in **Retro-2^cycl^**. The superimposition of the local minimum conformations of the two isomers shows nearly identical molecular shapes in terms of both steric and electrostatic properties (Figure 3), which explains the nearly equal potency of the two isomers for cell protection against ricin. This overlay also prompted us to synthesize and test 2-(5-methylthiophen-2-yl)-3-phenylquinazolin-4(3*H*)-one (**DA2MT**, Figure 1) as an achiral analog of **(±)-Retro-2^cycl^**. As apparent from Figure 3, the local minimum conformation of **DA2MT** derived at the HF/6-31G(d) level is nearly identical to those of the two isomers. Furthermore, **DA2MT** does not carry an aminal carbon, so it is chemically more stable than **(±)-Retro-2^cycl^**. The latter decomposes in the presence of (1s)-(+)-10-camphorsulfonic acid as described above. Accordingly, we synthesized **DA2MT** in quantitative yield through oxidation of **(±)-Retro-2^cycl^** using 2,3-dichloro-5,6-dicyano-1,4-benzoquinone rather than following a published procedure^15^ that uses potassium permanganate in acetone and has a solubility issue for **(±)-Retro-2^cycl^**.

### Cell-based assays of Retro-2^cycl^ Enantiomers and DA2MT

To determine the relative potencies of **(±)-Retro-2^cycl^**, **(+)-Retro-2^cycl^**, **(−)-Retro-2^cycl^**, and **DA2MT** in protecting cells against ricin, we examined protein synthesis inhibition by ricin in Vero cells by measuring [^35^S]-Met-incorporation. The concentrations used were based on the reported dose-response curves for ricin^7^. As shown in Figure 4, the presence of 2.5 and 5.0 μM each of the four inhibitors restored ~7 and ~12% of protein synthesis in the presence of 50 ng/mL ricin. Interestingly, all four inhibitors showed nearly equal potency of cell protection against ricin. This observation offers important insight into generalizing the structurally distinct chemicals that inhibit the intracellular transport of RIPs to be discussed below.

## Discussion

**Figure 2:**
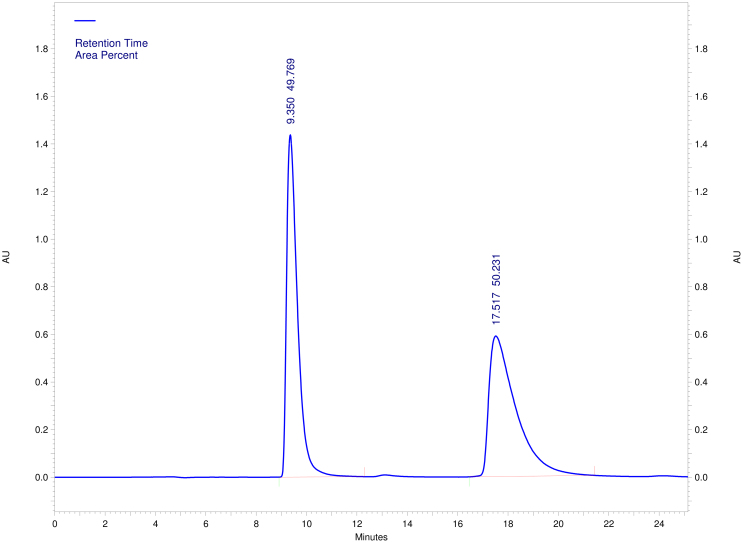
Chromatogram of high-performance liquid chromatography separation of (±)-Retro-2^cycl^ using the Lux™ Amylose-2 chiral column.

Substituted 2,3-dihydroquinazolin-4(1*H*)-ones have been well studied for various biological properties^8^,^16^,^17^,^18^,^19^,^20^,^21^,^22^,^23^. Racemization of the aminal stereo center in the substituted 2,3-dihydroquinazolin-4(1*H*)-ones has been reported^8^. One would suspect that the aminal stereo center in **Retro-2^cycl^** could be sensitive to racemization and possibly cause the lack of the stereo preference for cell protection that we observed. However, the **Retro-2^cycl^** structure has the substitution by a phenyl ring at its amide nitrogen atom. Theoretically this substitution lowers the propensity of the aminal stereo center for racemization. Indeed, through HPLC separation using a chiral column, we were able to separate **(±)-Retro-2^cycl^**. The optical activities and purities of the two enantiomers are demonstrated by the specific optical rotations and the HPLC chromatogram shown in Figure 2. In addition, we measured the rotations of the two enantiomers 14 months after the HPLC separation and found no racemization of the two isomers over the 14-month period. Furthermore, **DA2MT**, an achiral analog of **(±)-Retro-2^cycl^**, showed similar cell protection against ricin as the two isomers. These results preclude the possibility that racemization of the enantiomers led to the paucity of stereo preference for the cell protection against ricin.

**DA2MT** has been briefly reported to show no protection of HeLa cells against Shiga-like toxin 1, while a procedure for preparing **DA2MT** and the standard structure characterization have not been reported^24^. In this study, we found that **DA2MT** is as active as **Retro-2^cycl^** in protecting Vero cells against ricin. In view of its chemical stability and the paucity of chirality, **DA2MT** is a chemically improved analog of **Retro-2^cycl^** for cell protection against ricin and a useful lead for RIP antidote development as exemplified below.

**Figure 3:**
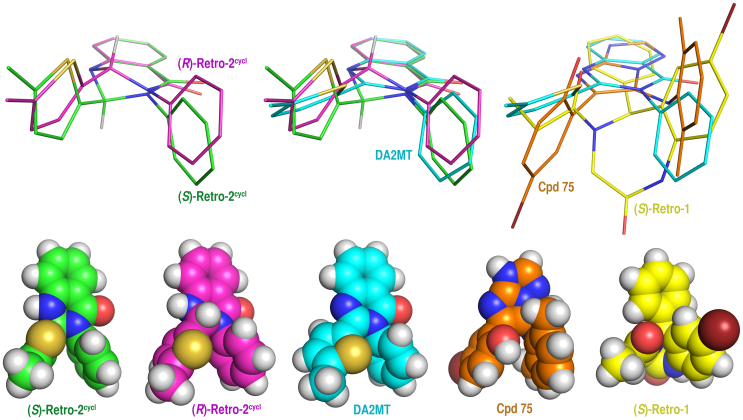
Local minimum conformations of structurally distinct RIP inhibitors derived at the HF/6-31G(d) level and their superimpositions. A (upper left): **(*S*)-Retro-2^cycl^** and **(*R*)-Retro-2^cycl^**; B (upper middle): **(*S*)-Retro-2^cycl^**, **(*R*)-Retro-2^cycl^**, and **DA2MT**; C (upper right): **DA2MT, (*S*)-Retro-1**, and **Cpd 75**; D (lower panel): local minimum conformations of **(*S*)-Retro-2^cycl^**, **(*R*)-Retro-2^cycl^**, **DA2MT, (*S*)-Retro-1**, and **Cpd 75** in sphere model. For clarity, all H atoms except for those at the chiral centers are undisplayed in the superimposed structures. The Br, S, O, N, and H atoms are colored in dark red, dark yellow, red, blue, and white, respectively. The C atoms in **(*S*)-Retro-2^cycl^**, **(*R*)-Retro-2^cycl^**, **DA2MT, Cpd 75**, and **(*S*)-Retro-1** are in green, magenta, cyan, orange, and yellow, respectively.

**Figure 4:**
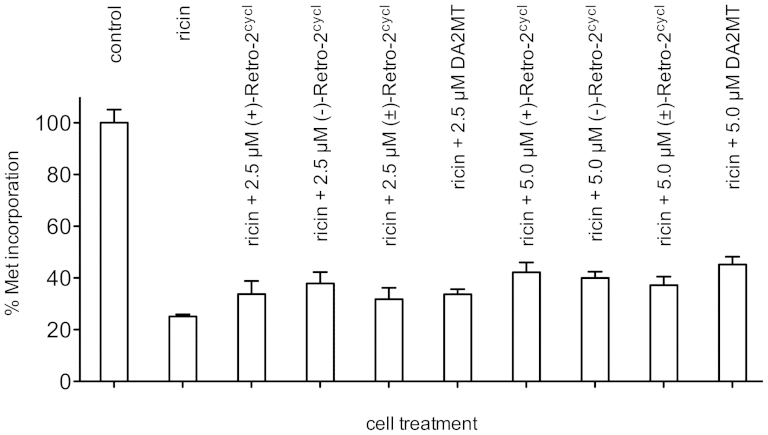
Cell protection against 50 ng/mL ricin by (±)-Retro-2^cycl^, (+)-Retro-2^cycl^, (−)-Retro-2^cycl^, and DA2MT in Vero cells.

As described above, the local minimum conformations of **(+)-Retro-2^cycl^**, **(−)-Retro-2^cycl^**, and **DA2MT** superimpose well (Figure 3). These conformations also superimpose well with the local minimum conformations of **(*S*)-Retro-1**^6^ and **Compound 75**^25^, both of which are effective intracellular RIP transport inhibitors (Figure 3). It is now well accepted that small molecules do not necessarily adopt their lowest potential energy conformations but do adopt local minimum conformations when binding to proteins^26^,^27^. All the chemical structures shown in Figure 3 are rigid. Their local minimum conformations were derived from *ab initio* calculations at the HF/6-31G(d) level and confirmed by frequency calculations using the same theory and basis set. Therefore the overlay shown in Figure 3 is reliable and strongly suggests that **(+)-Retro-2^cycl^**, **(−)-Retro-2^cycl^**, and **DA2MT**, **(*S*)-Retro-1**, and **Compound 75** (abbreviated as **Cpd 75**, Figure 1) share a common pharmacophore that targets the same protein critically involved in intracellular RIP transport. Structurally, this pharmacophore comprises a central aromatic ring with two substituents, one carrying an aromatic ring, and the other bearing an O or S atom attached to sp^2^ C atom(s). Detailed structural information is available from the Cartesian coordinates of the superimposed structures at their local-minimum conformations provided in Supplementary Information.

This pharmacophore unifies structurally distinct chemicals identified by different research laboratories calling attention to the possibility that **Retro-1**, **Retro-2^cycl^**, **DA2MT**, and **Compound 75** may have the same target site for their inhibition of RIP transport. It also offers new insights into lead identification and optimization for RIP antidote development to minimize the global health threat caused by RIPs. For example, focused combinatorial library synthesis to derivatize **DA2MT** with the guidance of the pharmacophore holds promise in identifying improved RIP transport inhibitors. Alternatively, in silico database screening for chemicals with the pharmacophore described above may result in new leads for RIP transport inhibitors. Reverse engineering with 20 essential amino acids for proteins with cavities that can accommodate the pharmacophore is now technically feasible with today's computing capability. Such reverse engineered proteins can thus offer guidance in lead optimization of RIP transport inhibitors, which may address the concern that the RIP transport inhibitors are undesirable because of the lack of structural information about the targets of current RIP transport inhibitor leads.

## Methods

### Separation of (±)-Retro-2^cycl^

About 10 mg of **(±)-Retro-2^cycl^** prepared according to our published procedure^7^ was separated using the Lux™ Amylose-2 chiral column on a Beckman Coulter HPLC System Gold 125P eluting with n-hexane/2-propanol (60/40) at a flow rate of 1.0 mL/min. The retention times of the plus and minus enantiomers are 9.350 and 17.517 minutes, respectively. The two fractions were collected and the eluent was removed in vacuo. The chemical identities of both isomers were confirmed by proton NMR analysis. The specific optical rotations of the two enantiomers are 118.7° (c = 0.21 in CHCl_3_) and −126.5° (c = 0.29 in CHCl_3_), respectively, which were measured using a Jasco DIP-370 Digital Polarimeter at 598 nm at room temperature with L = 0.5 dm.

### General description of chemical synthesis

All commercially available reagents were used as received. ^1^H NMR (400 MHz) and ^13^C NMR (100 MHz) spectra were recorded on a Mercury 400 spectrometer from Varian (Palo Alto, CA). Chemical shifts are reported in ppm using the solvent peak as an internal standard. Data are reported as follows: chemical shift, multiplicity (s = singlet, d = doublet, t = triplet, and m = multiplet), coupling constant, and integration. Low-resolution mass spectra (LRMS) were recorded using either a Hewlett Packard 5973 Mass Spectrometer with SIS Direct Insertion Probe (Palo Alto, CA) or a Waters ZQ/EMD 1000 Mass Spectrometer (Milford, MA). High-resolution mass spectra (HRMS) were obtained on a Bruker BioTOF II ESI. IR spectra were obtained on a ThermoNicolet Avatar 370 FT-IR (Waltham, MA) using a KBr pellet. Melting points were determined by using Mel-Temp II (Holliston, MA). Elemental Analysis was performed at NuMega (San Diego, CA).

### Synthesis of DA2MT (2-(5-methylthiophen-2-yl)-3-phenylquinazolin-4(3*H*)-one)

To a stirred solution of **Retro-2^cycl^** (64.0 mg, 0.20 mmol) in chloroform (5 mL) was added 2,3-dichloro-5,6-dicyano-1,4-benzoquinone (48.0 mg, 0.21 mmol) at room temperature. After 15 minutes of stirring, the reaction mixture was washed with saturated NaHCO_3_ solution (10 mL) to remove the remaining 2,3-dichloro-5,6-dicyano-1,4-benzoquinone and 4,5-dichloro-3,6-dihydroxyphthalonitrile as the by-product. The layers were separated, and the organic layer was dried over MgSO_4_, filtered, and concentrated to give 63.1 mg of pure product as a white solid in quantitative yield. Melting point 172–173.5°C; ^1^H NMR (400 MHz, CDCl_3_) δ 8.27 (d, *J* = 8.0 Hz, 1H), 7.78–7.72 (m, 2H), 7.54–7.51 (m, 3H), 7.44 (ddd, *J* = 8.0, 6.2, 2.0 Hz, 1H), 7.36–7.33 (m, 2H), 6.42 (d, *J* = 3.8 Hz, 1H), 6.04 (d, *J* = 3.8 Hz, 1H), and 2.40 (s, 3H); ^13^C NMR (100 MHz, CDCl_3_) δ 162.82, 148.85, 148.06, 146.34, 138.08, 135.78, 134.92, 131.86, 130.02, 129.79, 129.39, 127.56, 127.34, 126.83, 126.40, 120.46, and 15.56; IR (KBr) ν 3061, 2915, 1678, 1539, 768, and 700 cm^−1^; LRMS *m/z* 318 (100%, [M]^+^), HRMS-ESI *m/z* 319.0898 ([M + H]^+^, C_19_H_15_N_2_OS^+^ requires 319.0905). Anal. calcd for C_19_H_14_N_2_OS: C, 71.67; H, 4.43; N, 8.80. Found: C, 71.70; H, 4.80; N, 8.87.

### Computational studies

Different conformations of each inhibitor shown in Figure 3 were systematically generated by alternating the bulky groups at axial and equatorial positions and subsequently energy minimized with the MMX force field using the PCModel 91 program (Serena Software). These resulting conformations were subjected to energy minimization at the HF/6-31G(d) level using the Gaussian 98 program^28^. All the energy-minimized conformations at the HF/6-31G(d) level were checked for possible imaginary frequencies by subsequent frequency calculations using the same theory and basis set. The energy-minimized conformations with no imaginary frequencies were then manually superimposed using the Pair Fitting tool of the MacPyMOL V1.5.0 (Schrödinger LLC, Portland, OR), which led to the superimposed inhibitor structures shown in Figure 3.

### [^35^S]-Methionine incorporation assay

Vero cells were maintained in Dulbecco's modified Eagle medium with 10% fetal calf serum and 1 mM glutamine. The cells were resuspended after trypsin treatment at 4 × 10^4^ cells/mL in the same medium, and 0.5 mL of the medium was dispensed into 24-well plates. After 24 hours at 37°C and 5% CO_2_, the medium was changed to Dulbecco's modified Eagle medium without Met, Gln, or fetal calf serum and equilibrated for 1 hour. An inhibitor solution with a final dimethyl sulfoxide concentration of 0.5% was added to the medium at 25 hours. Ricin was added after 26 hours at varied concentrations. [^35^S]-Met was added 2 hours after ricin exposure. The [^35^S]-Met incorporation was terminated 30 minutes after the Met addition via medium removal and addition of 150 μL of 0.2 M aqueous KOH to dissolve cells, as described elsewhere^29^. Proteins were precipitated with 10% trichloroacetic acid, harvested on glass fiber filters, and counted. The control incorporation was determined after treatment with 0.5% dimethyl sulfoxide alone. Ricin was purchased from Vector Laboratories (Burlingame, CA).

## Author Contributions

Y.-P.P. and N.E.T. conceived and supervised the project; S.Y. designed and performed the chemical resolution studies; J.G.P. designed and performed chemical synthesis of DA2MT; J.N.K. performed the cell-based assays; Y.-P.P. designed and performed the computational studies; all authors analyzed the data; Y.-P.P. wrote the paper; all authors contributed with revisions.

## Supplementary Material

Supplementary Information Supplementary Information
