# Supplementary material for: Common Pharmacophore of Structurally Distinct Small-Molecule Inhibitors of Intracellular Retrograde Trafficking of Ribosome Inactivating Proteins
Source: Sci Rep. 2013 Dec 2;3:3397. doi: 10.1038/srep03397 (PMC3844963; doi:10.1038/srep03397)
Supplement: Supplementary Information [file srep03397-s1.pdf]

Supplementary Information

Common Pharmacophore of Structurally Distinct Small-Molecule Inhibitors of Intracellular Retrograde Trafficking of Ribosome Inactivating Proteins

Shichao Yu[1], Jewn Giew Park[1], Jennifer Nielsen Kahn[2], Nilgun E. Tumer[2,*], and Yuan-Ping Pang[1,*]

[1]Computer-Aided Molecular Design Laboratory, Mayo Clinic, Rochester, MN 55905, USA

[2]Department of Plant Biology and Pathology, School of Environmental and Biological Sciences, Rutgers University, New Brunswick, NJ 08901, USA

[*]Correspondence: pang@mayo.edu (Y.-P.P. for chemistry) and tumer@aesop.rutger.edu (N.E.T. for biology)

Page 2: Dataset S1. Cartesian coordinates of (*S*)-**Retro-2**^{cycl} that is energy minimized at the HF/6-31G(d) level.

Page 6: Dataset S2. Cartesian coordinates of (*R*)-**Retro-2**^{cycl} that is energy minimized at the HF/6-31G(d) level and superimposed over (*S*)-**Retro-2**^{cycl}.

Page 10: Dataset S3. Cartesian coordinates of **DA2MT** that is energy minimized at the HF/6-31G(d) level and superimposed over (*S*)-**Retro-2**^{cycl}.

Page 14: Dataset S4. Cartesian coordinates of **Cpd 75** that is energy minimized at the HF/6-31G(d) level and superimposed over (*S*)-**Retro-2**^{cycl}.

Page 19: Dataset S5. Cartesian coordinates of (*S*)-**Retro-1** that is energy minimized at the HF/6-31G(d) level and superimposed over (*S*)-**Retro-2**^{cycl}.

Dataset S1. Cartesian coordinates of **(*S*)-Retro-2^{cycl}** that is energy minimized at the HF/6-31G(d) level.

```
HETATM    1  N           1      -0.057  -0.141   0.013  0.00  0.00           N
HETATM    2  N           1       2.170  -0.936  -0.108  0.00  0.00           N
HETATM    3  C           1       0.040   0.100   1.369  0.00  0.00           C
HETATM    4  C           1       0.835  -1.113  -0.631  0.00  0.00           C
HETATM    5  C           1      -1.337   0.098  -0.592  0.00  0.00           C
HETATM    6  C           1       1.280  -0.377   2.030  0.00  0.00           C
HETATM    7  C           1      -2.335  -0.861  -0.522  0.00  0.00           C
HETATM    8  C           1      -1.576   1.295  -1.242  0.00  0.00           C
HETATM    9  C           1       0.882  -0.967  -2.127  0.00  0.00           C
HETATM   10  C           1       1.414  -0.268   3.410  0.00  0.00           C
HETATM   11  C           1       2.325  -0.886   1.267  0.00  0.00           C
HETATM   12  C           1      -3.563  -0.631  -1.118  0.00  0.00           C
HETATM   13  C           1      -2.807   1.528  -1.834  0.00  0.00           C
HETATM   14  C           1       0.350  -1.801  -3.036  0.00  0.00           C
HETATM   15  C           1       2.576  -0.669   4.034  0.00  0.00           C
HETATM   16  C           1       3.505  -1.283   1.893  0.00  0.00           C
HETATM   17  C           1      -3.800   0.565  -1.777  0.00  0.00           C
HETATM   18  C           1       1.290  -0.243  -4.479  0.00  0.00           C
HETATM   19  C           1       0.581  -1.384  -4.391  0.00  0.00           C
HETATM   20  C           1       3.619  -1.173   3.263  0.00  0.00           C
HETATM   21  C           1       1.720   0.484  -5.720  0.00  0.00           C
HETATM   22  O           1      -0.822   0.672   1.975  0.00  0.00           O
HETATM   23  S           1       1.705   0.356  -2.902  0.00  0.00           S
HETATM   24  H           1       0.465  -2.119  -0.423  0.00  0.00           H
HETATM   25  H           1       2.845  -1.493  -0.585  0.00  0.00           H
HETATM   26  H           1      -2.157  -1.780   0.008  0.00  0.00           H
HETATM   27  H           1      -0.800   2.037  -1.279  0.00  0.00           H
HETATM   28  H           1       0.593   0.138   3.968  0.00  0.00           H
HETATM   29  H           1      -4.334  -1.378  -1.061  0.00  0.00           H
HETATM   30  H           1      -2.988   2.461  -2.336  0.00  0.00           H
HETATM   31  H           1      -0.205  -2.683  -2.774  0.00  0.00           H
HETATM   32  H           1       2.679  -0.589   5.101  0.00  0.00           H
HETATM   33  H           1       4.320  -1.671   1.306  0.00  0.00           H
HETATM   34  H           1      -4.755   0.746  -2.237  0.00  0.00           H
HETATM   35  H           1       0.225  -1.926  -5.247  0.00  0.00           H
HETATM   36  H           1       4.532  -1.484   3.739  0.00  0.00           H
HETATM   37  H           1       1.374  -0.049  -6.597  0.00  0.00           H
HETATM   38  H           1       2.801   0.565  -5.777  0.00  0.00           H
HETATM   39  H           1       1.311   1.488  -5.751  0.00  0.00           H
CONECT    1    3    4    5
CONECT    2    4   11   25
CONECT    3    1    6
CONECT    3   22
CONECT    3   22
CONECT    4    1    2    9   24
CONECT    5    1
CONECT    5    7
CONECT    5    7
CONECT    5    7
CONECT    5    7
CONECT    5    8
CONECT    5    8
```

```
CONECT    5    8
CONECT    5    8
CONECT    6    3
CONECT    6   10
CONECT    6   10
CONECT    6   10
CONECT    6   10
CONECT    6   11
CONECT    6   11
CONECT    6   11
CONECT    6   11
CONECT    7    5
CONECT    7    5
CONECT    7    5
CONECT    7    5
CONECT    7   12
CONECT    7   12
CONECT    7   12
CONECT    7   12
CONECT    7   26
CONECT    8    5
CONECT    8    5
CONECT    8    5
CONECT    8    5
CONECT    8   13
CONECT    8   13
CONECT    8   13
CONECT    8   13
CONECT    8   27
CONECT    9    4
CONECT    9   14
CONECT    9   14
CONECT    9   14
CONECT    9   14
CONECT    9   23
CONECT    9   23
CONECT    9   23
CONECT    9   23
CONECT   10    6
CONECT   10    6
CONECT   10    6
CONECT   10    6
CONECT   10   15
CONECT   10   15
CONECT   10   15
CONECT   10   15
CONECT   10   28
CONECT   11    2
CONECT   11    6
CONECT   11    6
CONECT   11    6
CONECT   11    6
CONECT   11   16
CONECT   11   16
CONECT   11   16
```

```
CONECT   11   16
CONECT   12    7
CONECT   12    7
CONECT   12    7
CONECT   12    7
CONECT   12   17
CONECT   12   17
CONECT   12   17
CONECT   12   17
CONECT   12   29
CONECT   13    8
CONECT   13    8
CONECT   13    8
CONECT   13    8
CONECT   13   17
CONECT   13   17
CONECT   13   17
CONECT   13   17
CONECT   13   30
CONECT   14    9
CONECT   14    9
CONECT   14    9
CONECT   14    9
CONECT   14   19
CONECT   14   19
CONECT   14   19
CONECT   14   19
CONECT   14   31
CONECT   15   10
CONECT   15   10
CONECT   15   10
CONECT   15   10
CONECT   15   20
CONECT   15   20
CONECT   15   20
CONECT   15   20
CONECT   15   32
CONECT   16   11
CONECT   16   11
CONECT   16   11
CONECT   16   11
CONECT   16   20
CONECT   16   20
CONECT   16   20
CONECT   16   20
CONECT   16   33
CONECT   17   12
CONECT   17   12
CONECT   17   12
CONECT   17   12
CONECT   17   13
CONECT   17   13
CONECT   17   13
CONECT   17   13
CONECT   17   34
```

```
CONECT   18   19
CONECT   18   19
CONECT   18   19
CONECT   18   19
CONECT   18   21
CONECT   18   23
CONECT   18   23
CONECT   18   23
CONECT   18   23
CONECT   19   14
CONECT   19   14
CONECT   19   14
CONECT   19   14
CONECT   19   18
CONECT   19   18
CONECT   19   18
CONECT   19   18
CONECT   19   35
CONECT   20   15
CONECT   20   15
CONECT   20   15
CONECT   20   15
CONECT   20   16
CONECT   20   16
CONECT   20   16
CONECT   20   16
CONECT   20   36
CONECT   21   18   37   38   39
CONECT   22    3
CONECT   22    3
CONECT   23    9
CONECT   23    9
CONECT   23    9
CONECT   23    9
CONECT   23   18
CONECT   23   18
CONECT   23   18
CONECT   23   18
CONECT   24    4
CONECT   25    2
CONECT   26    7
CONECT   27    8
CONECT   28   10
CONECT   29   12
CONECT   30   13
CONECT   31   14
CONECT   32   15
CONECT   33   16
CONECT   34   17
CONECT   35   19
CONECT   36   20
CONECT   37   21
CONECT   38   21
CONECT   39   21
END
```

Dataset S2. Cartesian coordinates of **(*R*)-Retro-2^{cycl}** that is energy minimized at the HF/6-31G(d) level and superimposed over **(*S*)-Retro-2^{cycl}**.

```
HETATM    1  N             1       0.000   0.000   0.000  0.00  0.00           N
HETATM    2  N             1       2.160  -0.959  -0.106  0.00  0.00           N
HETATM    3  C             1       0.000   0.000   1.379  0.00  0.00           C
HETATM    4  C             1       1.275   0.000  -0.729  0.00  0.00           C
HETATM    5  C             1      -1.157   0.547  -0.648  0.00  0.00           C
HETATM    6  C             1       1.282  -0.373   2.029  0.00  0.00           C
HETATM    7  C             1      -2.107  -0.302  -1.189  0.00  0.00           C
HETATM    8  C             1      -1.330   1.920  -0.733  0.00  0.00           C
HETATM    9  C             1       1.126  -0.378  -2.183  0.00  0.00           C
HETATM   10  C             1       1.404  -0.294   3.413  0.00  0.00           C
HETATM   11  C             1       2.338  -0.855   1.264  0.00  0.00           C
HETATM   12  C             1      -3.222   0.220  -1.824  0.00  0.00           C
HETATM   13  C             1      -2.444   2.441  -1.370  0.00  0.00           C
HETATM   14  C             1       0.953  -1.609  -2.696  0.00  0.00           C
HETATM   15  C             1       2.570  -0.684   4.036  0.00  0.00           C
HETATM   16  C             1       3.515  -1.260   1.891  0.00  0.00           C
HETATM   17  C             1      -3.390   1.592  -1.918  0.00  0.00           C
HETATM   18  C             1       1.022  -0.393  -4.678  0.00  0.00           C
HETATM   19  C             1       0.888  -1.616  -4.130  0.00  0.00           C
HETATM   20  C             1       3.621  -1.170   3.264  0.00  0.00           C
HETATM   21  C             1       1.003  -0.021  -6.132  0.00  0.00           C
HETATM   22  O             1      -0.980   0.266   2.016  0.00  0.00           O
HETATM   23  S             1       1.229   0.810  -3.445  0.00  0.00           S
HETATM   24  H             1       1.701   1.004  -0.676  0.00  0.00           H
HETATM   25  H             1       2.999  -1.094  -0.627  0.00  0.00           H
HETATM   26  H             1      -1.968  -1.363  -1.107  0.00  0.00           H
HETATM   27  H             1      -0.603   2.577  -0.291  0.00  0.00           H
HETATM   28  H             1       0.567   0.071   3.976  0.00  0.00           H
HETATM   29  H             1      -3.957  -0.444  -2.241  0.00  0.00           H
HETATM   30  H             1      -2.573   3.507  -1.431  0.00  0.00           H
HETATM   31  H             1       0.875  -2.490  -2.089  0.00  0.00           H
HETATM   32  H             1       2.666  -0.619   5.104  0.00  0.00           H
HETATM   33  H             1       4.332  -1.640   1.304  0.00  0.00           H
HETATM   34  H             1      -4.257   1.996  -2.411  0.00  0.00           H
HETATM   35  H             1       0.748  -2.508  -4.712  0.00  0.00           H
HETATM   36  H             1       4.534  -1.481   3.740  0.00  0.00           H
HETATM   37  H             1       0.872  -0.911  -6.737  0.00  0.00           H
HETATM   38  H             1       1.930   0.457  -6.433  0.00  0.00           H
HETATM   39  H             1       0.190   0.662  -6.356  0.00  0.00           H
CONECT    1    3    4    5
CONECT    2    4   11   25
CONECT    3    1    6
CONECT    3   22
CONECT    3   22
CONECT    4    1    2    9   24
CONECT    5    1
CONECT    5    7
CONECT    5    7
CONECT    5    7
CONECT    5    7
```

```
CONECT    5    8
CONECT    5    8
CONECT    5    8
CONECT    5    8
CONECT    6    3
CONECT    6   10
CONECT    6   10
CONECT    6   10
CONECT    6   10
CONECT    6   11
CONECT    6   11
CONECT    6   11
CONECT    6   11
CONECT    7    5
CONECT    7    5
CONECT    7    5
CONECT    7    5
CONECT    7   12
CONECT    7   12
CONECT    7   12
CONECT    7   12
CONECT    7   26
CONECT    8    5
CONECT    8    5
CONECT    8    5
CONECT    8    5
CONECT    8   13
CONECT    8   13
CONECT    8   13
CONECT    8   13
CONECT    8   27
CONECT    9    4
CONECT    9   14
CONECT    9   14
CONECT    9   14
CONECT    9   14
CONECT    9   23
CONECT    9   23
CONECT    9   23
CONECT    9   23
CONECT   10    6
CONECT   10    6
CONECT   10    6
CONECT   10    6
CONECT   10   15
CONECT   10   15
CONECT   10   15
CONECT   10   15
CONECT   10   28
CONECT   11    2
CONECT   11    6
CONECT   11    6
CONECT   11    6
CONECT   11    6
CONECT   11   16
```

```
CONECT   11   16
CONECT   11   16
CONECT   11   16
CONECT   12    7
CONECT   12    7
CONECT   12    7
CONECT   12    7
CONECT   12   17
CONECT   12   17
CONECT   12   17
CONECT   12   17
CONECT   12   29
CONECT   13    8
CONECT   13    8
CONECT   13    8
CONECT   13    8
CONECT   13   17
CONECT   13   17
CONECT   13   17
CONECT   13   17
CONECT   13   30
CONECT   14    9
CONECT   14    9
CONECT   14    9
CONECT   14    9
CONECT   14   19
CONECT   14   19
CONECT   14   19
CONECT   14   19
CONECT   14   31
CONECT   15   10
CONECT   15   10
CONECT   15   10
CONECT   15   10
CONECT   15   20
CONECT   15   20
CONECT   15   20
CONECT   15   20
CONECT   15   32
CONECT   16   11
CONECT   16   11
CONECT   16   11
CONECT   16   11
CONECT   16   20
CONECT   16   20
CONECT   16   20
CONECT   16   20
CONECT   16   33
CONECT   17   12
CONECT   17   12
CONECT   17   12
CONECT   17   12
CONECT   17   13
CONECT   17   13
CONECT   17   13
```

```
CONECT   17   13
CONECT   17   34
CONECT   18   19
CONECT   18   19
CONECT   18   19
CONECT   18   19
CONECT   18   21
CONECT   18   23
CONECT   18   23
CONECT   18   23
CONECT   18   23
CONECT   19   14
CONECT   19   14
CONECT   19   14
CONECT   19   14
CONECT   19   18
CONECT   19   18
CONECT   19   18
CONECT   19   18
CONECT   19   35
CONECT   20   15
CONECT   20   15
CONECT   20   15
CONECT   20   15
CONECT   20   16
CONECT   20   16
CONECT   20   16
CONECT   20   16
CONECT   20   36
CONECT   21   18   37   38   39
CONECT   22    3
CONECT   22    3
CONECT   23    9
CONECT   23    9
CONECT   23    9
CONECT   23    9
CONECT   23   18
CONECT   23   18
CONECT   23   18
CONECT   23   18
CONECT   24    4
CONECT   25    2
CONECT   26    7
CONECT   27    8
CONECT   28   10
CONECT   29   12
CONECT   30   13
CONECT   31   14
CONECT   32   15
CONECT   33   16
CONECT   34   17
CONECT   35   19
CONECT   36   20
CONECT   37   21
CONECT   38   21
```

```
CONECT   39   21
END
```

Dataset S3. Cartesian coordinates of **DA2MT** that is energy minimized at the HF/6-31G(d) level and superimposed over **(*S*)-Retro-2**$^{\mathbf{cycl}}$**.**

```
HETATM    1  N             1        0.045  -0.032   0.015  0.00  0.00            N
HETATM    2  N             1        2.198  -0.957  -0.099  0.00  0.00            N
HETATM    3  C             1        0.088   0.179   1.400  0.00  0.00            C
HETATM    4  C             1        1.128  -0.571  -0.668  0.00  0.00            C
HETATM    5  C             1       -1.223   0.215  -0.620  0.00  0.00            C
HETATM    6  C             1        1.320  -0.270   2.049  0.00  0.00            C
HETATM    7  C             1       -2.017  -0.852  -0.998  0.00  0.00            C
HETATM    8  C             1       -1.653   1.516  -0.818  0.00  0.00            C
HETATM    9  C             1        1.083  -0.740  -2.142  0.00  0.00            C
HETATM   10  C             1        1.475  -0.147   3.433  0.00  0.00            C
HETATM   11  C             1        2.316  -0.843   1.272  0.00  0.00            C
HETATM   12  C             1       -3.238  -0.617  -1.609  0.00  0.00            C
HETATM   13  C             1       -2.877   1.747  -1.420  0.00  0.00            C
HETATM   14  C             1        1.658  -1.783  -2.774  0.00  0.00            C
HETATM   15  C             1        2.624  -0.598   4.032  0.00  0.00            C
HETATM   16  C             1        3.487  -1.300   1.890  0.00  0.00            C
HETATM   17  C             1       -3.668   0.681  -1.822  0.00  0.00            C
HETATM   18  C             1        1.038  -0.539  -4.629  0.00  0.00            C
HETATM   19  C             1        1.619  -1.677  -4.199  0.00  0.00            C
HETATM   20  C             1        3.632  -1.177   3.249  0.00  0.00            C
HETATM   21  C             1        0.827  -0.084  -6.045  0.00  0.00            C
HETATM   22  O             1       -0.836   0.671   1.978  0.00  0.00            O
HETATM   23  S             1        0.491   0.422  -3.297  0.00  0.00            S
HETATM   24  H             1       -1.678  -1.857  -0.822  0.00  0.00            H
HETATM   25  H             1       -1.038   2.334  -0.496  0.00  0.00            H
HETATM   26  H             1        0.682   0.299   4.003  0.00  0.00            H
HETATM   27  H             1       -3.851  -1.447  -1.910  0.00  0.00            H
HETATM   28  H             1       -3.212   2.756  -1.575  0.00  0.00            H
HETATM   29  H             1        2.114  -2.597  -2.245  0.00  0.00            H
HETATM   30  H             1        2.755  -0.508   5.095  0.00  0.00            H
HETATM   31  H             1        4.255  -1.736   1.279  0.00  0.00            H
HETATM   32  H             1       -4.617   0.864  -2.292  0.00  0.00            H
HETATM   33  H             1        2.017  -2.424  -4.861  0.00  0.00            H
HETATM   34  H             1        4.533  -1.528   3.721  0.00  0.00            H
HETATM   35  H             1        1.232  -0.820  -6.730  0.00  0.00            H
HETATM   36  H             1        1.323   0.861  -6.234  0.00  0.00            H
HETATM   37  H             1       -0.227   0.041  -6.267  0.00  0.00            H
CONECT    1    3
CONECT    1    3
CONECT    1    3
CONECT    1    3
CONECT    1    4
CONECT    1    4
CONECT    1    4
CONECT    1    4
CONECT    1    5
CONECT    2    4
CONECT    2    4
CONECT    2    4
```

```
CONECT    2    4
CONECT    2   11
CONECT    2   11
CONECT    2   11
CONECT    2   11
CONECT    3    1
CONECT    3    1
CONECT    3    1
CONECT    3    1
CONECT    3    6
CONECT    3    6
CONECT    3    6
CONECT    3    6
CONECT    3   22
CONECT    3   22
CONECT    4    1
CONECT    4    1
CONECT    4    1
CONECT    4    1
CONECT    4    2
CONECT    4    2
CONECT    4    2
CONECT    4    2
CONECT    4    9
CONECT    5    1
CONECT    5    7
CONECT    5    7
CONECT    5    7
CONECT    5    7
CONECT    5    8
CONECT    5    8
CONECT    5    8
CONECT    5    8
CONECT    6    3
CONECT    6    3
CONECT    6    3
CONECT    6    3
CONECT    6   10
CONECT    6   10
CONECT    6   10
CONECT    6   10
CONECT    6   11
CONECT    6   11
CONECT    6   11
CONECT    6   11
CONECT    7    5
CONECT    7    5
CONECT    7    5
CONECT    7    5
CONECT    7   12
CONECT    7   12
CONECT    7   12
CONECT    7   12
CONECT    7   24
CONECT    8    5
```

```
CONECT    8    5
CONECT    8    5
CONECT    8    5
CONECT    8   13
CONECT    8   13
CONECT    8   13
CONECT    8   13
CONECT    8   25
CONECT    9    4
CONECT    9   14
CONECT    9   14
CONECT    9   14
CONECT    9   14
CONECT    9   23
CONECT    9   23
CONECT    9   23
CONECT    9   23
CONECT   10    6
CONECT   10    6
CONECT   10    6
CONECT   10    6
CONECT   10   15
CONECT   10   15
CONECT   10   15
CONECT   10   15
CONECT   10   26
CONECT   11    2
CONECT   11    2
CONECT   11    2
CONECT   11    2
CONECT   11    6
CONECT   11    6
CONECT   11    6
CONECT   11    6
CONECT   11   16
CONECT   11   16
CONECT   11   16
CONECT   11   16
CONECT   12    7
CONECT   12    7
CONECT   12    7
CONECT   12    7
CONECT   12   17
CONECT   12   17
CONECT   12   17
CONECT   12   17
CONECT   12   27
CONECT   13    8
CONECT   13    8
CONECT   13    8
CONECT   13    8
CONECT   13   17
CONECT   13   17
CONECT   13   17
CONECT   13   17
```

```
CONECT   13   28
CONECT   14    9
CONECT   14    9
CONECT   14    9
CONECT   14    9
CONECT   14   19
CONECT   14   19
CONECT   14   19
CONECT   14   19
CONECT   14   29
CONECT   15   10
CONECT   15   10
CONECT   15   10
CONECT   15   10
CONECT   15   20
CONECT   15   20
CONECT   15   20
CONECT   15   20
CONECT   15   30
CONECT   16   11
CONECT   16   11
CONECT   16   11
CONECT   16   11
CONECT   16   20
CONECT   16   20
CONECT   16   20
CONECT   16   20
CONECT   16   31
CONECT   17   12
CONECT   17   12
CONECT   17   12
CONECT   17   12
CONECT   17   13
CONECT   17   13
CONECT   17   13
CONECT   17   13
CONECT   17   32
CONECT   18   19
CONECT   18   19
CONECT   18   19
CONECT   18   19
CONECT   18   21
CONECT   18   23
CONECT   18   23
CONECT   18   23
CONECT   18   23
CONECT   19   14
CONECT   19   14
CONECT   19   14
CONECT   19   14
CONECT   19   18
CONECT   19   18
CONECT   19   18
CONECT   19   18
CONECT   19   33
```

```
CONECT   20   15
CONECT   20   15
CONECT   20   15
CONECT   20   15
CONECT   20   16
CONECT   20   16
CONECT   20   16
CONECT   20   16
CONECT   20   34
CONECT   21   18   35   36   37
CONECT   22    3
CONECT   22    3
CONECT   23    9
CONECT   23    9
CONECT   23    9
CONECT   23    9
CONECT   23   18
CONECT   23   18
CONECT   23   18
CONECT   23   18
CONECT   24    7
CONECT   25    8
CONECT   26   10
CONECT   27   12
CONECT   28   13
CONECT   29   14
CONECT   30   15
CONECT   31   16
CONECT   32   17
CONECT   33   19
CONECT   34   20
CONECT   35   21
CONECT   36   21
CONECT   37   21
END
```

Dataset S4. Cartesian coordinates of **Cpd 75** that is energy minimized at the HF/6-31G(d) level and superimposed over **(*S*)-Retro-2^{cycl}.**

```
HETATM    1  N            1       1.023   0.203   2.745  0.00  0.00           N
HETATM    2  N            1       1.090  -0.276   1.471  0.00  0.00           N
HETATM    3  N            1       2.945  -0.835   2.375  0.00  0.00           N
HETATM    4  N            1       2.325  -1.387   0.015  0.00  0.00           N
HETATM    5  N            1      -0.968   0.202   0.271  0.00  0.00           N
HETATM    6  C            1       2.145  -0.149   3.247  0.00  0.00           C
HETATM    7  C            1       2.231  -0.911   1.209  0.00  0.00           C
HETATM    8  C            1       0.319  -0.334   0.318  0.00  0.00           C
HETATM    9  C            1       1.113  -1.025  -0.550  0.00  0.00           C
HETATM   10  C            1       0.790  -1.426  -1.942  0.00  0.00           C
HETATM   11  C            1      -1.375   1.147  -0.705  0.00  0.00           C
HETATM   12  C            1       0.425  -2.738  -2.209  0.00  0.00           C
HETATM   13  C            1       0.859  -0.524  -3.003  0.00  0.00           C
HETATM   14  C            1      -0.754   2.399  -0.768  0.00  0.00           C
HETATM   15  C            1      -2.441   0.838  -1.557  0.00  0.00           C
```

```
HETATM   16  C            1       0.104  -3.136  -3.493  0.00  0.00           C
HETATM   17  C            1       0.534  -0.930  -4.289  0.00  0.00           C
HETATM   18  C            1      -1.188   3.309  -1.721  0.00  0.00           C
HETATM   19  C            1      -2.858   1.785  -2.484  0.00  0.00           C
HETATM   20  C            1      -3.142  -0.500  -1.486  0.00  0.00           C
HETATM   21  C            1       0.366   2.776   0.173  0.00  0.00           C
HETATM   22  C            1       0.150  -2.235  -4.538  0.00  0.00           C
HETATM   23  C            1      -2.233   3.013  -2.579  0.00  0.00           C
HETATM   24  O            1       1.277   0.733  -2.746  0.00  0.00           O
HETATM   25 Br            1      -0.410  -4.936  -3.821  0.00  0.00          Br
HETATM   26  H            1       2.438   0.069   4.251  0.00  0.00           H
HETATM   27  H            1       3.852  -1.203   2.545  0.00  0.00           H
HETATM   28  H            1      -1.675  -0.458   0.508  0.00  0.00           H
HETATM   29  H            1       0.387  -3.445  -1.403  0.00  0.00           H
HETATM   30  H            1       0.594  -0.227  -5.100  0.00  0.00           H
HETATM   31  H            1       0.931   1.342  -3.383  0.00  0.00           H
HETATM   32  H            1      -0.713   4.274  -1.773  0.00  0.00           H
HETATM   33  H            1      -3.679   1.550  -3.140  0.00  0.00           H
HETATM   34  H            1      -2.443  -1.325  -1.581  0.00  0.00           H
HETATM   35  H            1      -3.868  -0.591  -2.285  0.00  0.00           H
HETATM   36  H            1      -3.681  -0.629  -0.550  0.00  0.00           H
HETATM   37  H            1       0.518   3.849   0.163  0.00  0.00           H
HETATM   38  H            1       0.142   2.472   1.189  0.00  0.00           H
HETATM   39  H            1       1.298   2.303  -0.116  0.00  0.00           H
HETATM   40  H            1      -0.101  -2.545  -5.534  0.00  0.00           H
HETATM   41  H            1      -2.564   3.737  -3.302  0.00  0.00           H
CONECT    1    2
CONECT    1    2
CONECT    1    2
CONECT    1    2
CONECT    1    6
CONECT    1    6
CONECT    1    6
CONECT    1    6
CONECT    2    1
CONECT    2    1
CONECT    2    1
CONECT    2    1
CONECT    2    7
CONECT    2    7
CONECT    2    7
CONECT    2    7
CONECT    2    8
CONECT    2    8
CONECT    2    8
CONECT    2    8
CONECT    3    6
CONECT    3    6
CONECT    3    6
CONECT    3    6
CONECT    3    7
CONECT    3    7
CONECT    3    7
CONECT    3    7
CONECT    3   27
```

```
CONECT    4    7
CONECT    4    7
CONECT    4    7
CONECT    4    7
CONECT    4    9
CONECT    4    9
CONECT    4    9
CONECT    4    9
CONECT    5    8   11   28
CONECT    6    1
CONECT    6    1
CONECT    6    1
CONECT    6    1
CONECT    6    3
CONECT    6    3
CONECT    6    3
CONECT    6    3
CONECT    6   26
CONECT    7    2
CONECT    7    2
CONECT    7    2
CONECT    7    2
CONECT    7    3
CONECT    7    3
CONECT    7    3
CONECT    7    3
CONECT    7    4
CONECT    7    4
CONECT    7    4
CONECT    7    4
CONECT    8    2
CONECT    8    2
CONECT    8    2
CONECT    8    2
CONECT    8    5
CONECT    8    9
CONECT    8    9
CONECT    8    9
CONECT    8    9
CONECT    9    4
CONECT    9    4
CONECT    9    4
CONECT    9    4
CONECT    9    8
CONECT    9    8
CONECT    9    8
CONECT    9    8
CONECT    9   10
CONECT   10    9
CONECT   10   12
CONECT   10   12
CONECT   10   12
CONECT   10   12
CONECT   10   13
CONECT   10   13
```

```
CONECT   10   13
CONECT   10   13
CONECT   11    5
CONECT   11   14
CONECT   11   14
CONECT   11   14
CONECT   11   14
CONECT   11   15
CONECT   11   15
CONECT   11   15
CONECT   11   15
CONECT   12   10
CONECT   12   10
CONECT   12   10
CONECT   12   10
CONECT   12   16
CONECT   12   16
CONECT   12   16
CONECT   12   16
CONECT   12   29
CONECT   13   10
CONECT   13   10
CONECT   13   10
CONECT   13   10
CONECT   13   17
CONECT   13   17
CONECT   13   17
CONECT   13   17
CONECT   13   24
CONECT   14   11
CONECT   14   11
CONECT   14   11
CONECT   14   11
CONECT   14   18
CONECT   14   18
CONECT   14   18
CONECT   14   18
CONECT   14   21
CONECT   15   11
CONECT   15   11
CONECT   15   11
CONECT   15   11
CONECT   15   19
CONECT   15   19
CONECT   15   19
CONECT   15   19
CONECT   15   20
CONECT   16   12
CONECT   16   12
CONECT   16   12
CONECT   16   12
CONECT   16   22
CONECT   16   22
CONECT   16   22
CONECT   16   22
```

```
CONECT   16   25
CONECT   17   13
CONECT   17   13
CONECT   17   13
CONECT   17   13
CONECT   17   22
CONECT   17   22
CONECT   17   22
CONECT   17   22
CONECT   17   30
CONECT   18   14
CONECT   18   14
CONECT   18   14
CONECT   18   14
CONECT   18   23
CONECT   18   23
CONECT   18   23
CONECT   18   23
CONECT   18   32
CONECT   19   15
CONECT   19   15
CONECT   19   15
CONECT   19   15
CONECT   19   23
CONECT   19   23
CONECT   19   23
CONECT   19   23
CONECT   19   33
CONECT   20   15   34   35   36
CONECT   21   14   37   38   39
CONECT   22   16
CONECT   22   16
CONECT   22   16
CONECT   22   16
CONECT   22   17
CONECT   22   17
CONECT   22   17
CONECT   22   17
CONECT   22   40
CONECT   23   18
CONECT   23   18
CONECT   23   18
CONECT   23   18
CONECT   23   19
CONECT   23   19
CONECT   23   19
CONECT   23   19
CONECT   23   41
CONECT   24   13   31
CONECT   25   16
CONECT   26    6
CONECT   27    3
CONECT   28    5
CONECT   29   12
CONECT   30   17
```

```
CONECT   31   24
CONECT   32   18
CONECT   33   19
CONECT   34   20
CONECT   35   20
CONECT   36   20
CONECT   37   21
CONECT   38   21
CONECT   39   21
CONECT   40   22
CONECT   41   23
END
```

Dataset S5. Cartesian coordinates of **(*S*)-Retro-1** that is energy minimized at the HF/6-31G(d) level and superimposed over **(*S*)-Retro-2^{cycl}**.

```
HETATM    1  N            1       -2.734  -1.342  -1.020  0.00  0.00           N
HETATM    2  N            1        0.159  -1.747  -1.121  0.00  0.00           N
HETATM    3  C            1       -2.216  -0.058  -0.728  0.00  0.00           C
HETATM    4  C            1       -2.220  -2.565  -1.359  0.00  0.00           C
HETATM    5  C            1       -3.048   1.020  -1.009  0.00  0.00           C
HETATM    6  C            1       -0.971   0.166  -0.141  0.00  0.00           C
HETATM    7  C            1       -0.725  -2.863  -1.342  0.00  0.00           C
HETATM    8  C            1       -2.655   2.312  -0.724  0.00  0.00           C
HETATM    9  C            1       -0.572   1.468   0.112  0.00  0.00           C
HETATM   10  C            1       -0.104  -1.051   0.145  0.00  0.00           C
HETATM   11  C            1       -1.411   2.528  -0.168  0.00  0.00           C
HETATM   12  C            1        0.805  -1.083  -2.137  0.00  0.00           C
HETATM   13  C            1        1.090  -0.854   1.074  0.00  0.00           C
HETATM   14  C            1        0.843  -1.734  -3.513  0.00  0.00           C
HETATM   15  C            1        2.400  -1.141   0.723  0.00  0.00           C
HETATM   16  C            1        0.826  -0.450   2.381  0.00  0.00           C
HETATM   17  C            1        1.688  -0.936  -4.500  0.00  0.00           C
HETATM   18  C            1        3.423  -1.011   1.652  0.00  0.00           C
HETATM   19  C            1        1.844  -0.314   3.306  0.00  0.00           C
HETATM   20  C            1        3.153  -0.594   2.943  0.00  0.00           C
HETATM   21  O            1       -2.966  -3.455  -1.654  0.00  0.00           O
HETATM   22  O            1        1.349  -0.034  -1.934  0.00  0.00           O
HETATM   23 Br            1       -0.844   4.299   0.207  0.00  0.00          Br
HETATM   24  H            1       -3.727  -1.368  -1.115  0.00  0.00           H
HETATM   25  H            1       -4.009   0.847  -1.459  0.00  0.00           H
HETATM   26  H            1       -0.514  -3.364  -2.270  0.00  0.00           H
HETATM   27  H            1       -0.582  -3.603  -0.562  0.00  0.00           H
HETATM   28  H            1       -3.307   3.136  -0.943  0.00  0.00           H
HETATM   29  H            1        0.399   1.650   0.527  0.00  0.00           H
HETATM   30  H            1       -0.747  -1.735   0.688  0.00  0.00           H
HETATM   31  H            1       -0.173  -1.830  -3.886  0.00  0.00           H
HETATM   32  H            1        1.231  -2.744  -3.419  0.00  0.00           H
HETATM   33  H            1        2.637  -1.457  -0.271  0.00  0.00           H
HETATM   34  H            1       -0.187  -0.238   2.680  0.00  0.00           H
HETATM   35  H            1        1.299   0.067  -4.622  0.00  0.00           H
HETATM   36  H            1        1.686  -1.428  -5.466  0.00  0.00           H
HETATM   37  H            1        2.711  -0.851  -4.159  0.00  0.00           H
HETATM   38  H            1        4.432  -1.233   1.359  0.00  0.00           H
```

```
HETATM   39  H            1       1.616   0.003   4.308  0.00  0.00              H
HETATM   40  H            1       3.948  -0.493   3.659  0.00  0.00              H
CONECT    1    3    4   24
CONECT    2    7   10   12
CONECT    3    1
CONECT    3    5
CONECT    3    5
CONECT    3    5
CONECT    3    5
CONECT    3    6
CONECT    3    6
CONECT    3    6
CONECT    3    6
CONECT    4    1    7
CONECT    4   21
CONECT    4   21
CONECT    5    3
CONECT    5    3
CONECT    5    3
CONECT    5    3
CONECT    5    8
CONECT    5    8
CONECT    5    8
CONECT    5    8
CONECT    5   25
CONECT    6    3
CONECT    6    3
CONECT    6    3
CONECT    6    3
CONECT    6    9
CONECT    6    9
CONECT    6    9
CONECT    6    9
CONECT    6   10
CONECT    7    2    4   26   27
CONECT    8    5
CONECT    8    5
CONECT    8    5
CONECT    8    5
CONECT    8   11
CONECT    8   11
CONECT    8   11
CONECT    8   11
CONECT    8   28
CONECT    9    6
CONECT    9    6
CONECT    9    6
CONECT    9    6
CONECT    9   11
CONECT    9   11
CONECT    9   11
CONECT    9   11
CONECT    9   29
CONECT   10    2    6   13   30
CONECT   11    8
```

```
CONECT   11    8
CONECT   11    8
CONECT   11    8
CONECT   11    9
CONECT   11    9
CONECT   11    9
CONECT   11    9
CONECT   11   23
CONECT   12    2   14
CONECT   12   22
CONECT   12   22
CONECT   13   10
CONECT   13   15
CONECT   13   15
CONECT   13   15
CONECT   13   15
CONECT   13   16
CONECT   13   16
CONECT   13   16
CONECT   13   16
CONECT   14   12   17   31   32
CONECT   15   13
CONECT   15   13
CONECT   15   13
CONECT   15   13
CONECT   15   18
CONECT   15   18
CONECT   15   18
CONECT   15   18
CONECT   15   33
CONECT   16   13
CONECT   16   13
CONECT   16   13
CONECT   16   13
CONECT   16   19
CONECT   16   19
CONECT   16   19
CONECT   16   19
CONECT   16   34
CONECT   17   14   35   36   37
CONECT   18   15
CONECT   18   15
CONECT   18   15
CONECT   18   15
CONECT   18   20
CONECT   18   20
CONECT   18   20
CONECT   18   20
CONECT   18   38
CONECT   19   16
CONECT   19   16
CONECT   19   16
CONECT   19   16
CONECT   19   20
CONECT   19   20
```

```
CONECT   19   20
CONECT   19   20
CONECT   19   39
CONECT   20   18
CONECT   20   18
CONECT   20   18
CONECT   20   18
CONECT   20   19
CONECT   20   19
CONECT   20   19
CONECT   20   19
CONECT   20   40
CONECT   21    4
CONECT   21    4
CONECT   22   12
CONECT   22   12
CONECT   23   11
CONECT   24    1
CONECT   25    5
CONECT   26    7
CONECT   27    7
CONECT   28    8
CONECT   29    9
CONECT   30   10
CONECT   31   14
CONECT   32   14
CONECT   33   15
CONECT   34   16
CONECT   35   17
CONECT   36   17
CONECT   37   17
CONECT   38   18
CONECT   39   19
CONECT   40   20
END
```
